# Time Optimization of Seed-Mediated Gold Nanotriangle Synthesis Based on Kinetic Studies

**DOI:** 10.3390/nano11041049

**Published:** 2021-04-20

**Authors:** Ekaterina Podlesnaia, Andrea Csáki, Wolfgang Fritzsche

**Affiliations:** Department of Nanobiophotonics, Leibniz Institute of Photonic Technology (IPHT), Albert-Einstein-Straße 9, 07745 Jena, Germany; ekaterina.podlesnaia@leibniz-ipht.de (E.P.); andrea.csaki@leibniz-ipht.de (A.C.)

**Keywords:** gold nanoprisms, gold nanotriangles, shape anisotropy, triangular gold, kinetics, seed-mediated synthesis, localized surface plasmon resonance, plasmonic nanoparticles, time-resolved UV–VIS spectroscopy

## Abstract

The synthesis of shape-anisotropic plasmonic nanoparticles such as gold nanotriangles is of increasing interest. These particles have a high potential for applications due to their notable optical properties. A key challenge of the synthesis is usually the low reproducibility. Even the optimized seed-based methods often lack in the synthesis yield or are labor- and time-consuming. In this work, a seed-mediated synthesis with high reproducibility is replicated in order to determine the necessary reaction time for each step. Online monitoring of the reaction mixtures by UV–VIS spectroscopy is used as a powerful tool to track the evolution of the synthesis. The kinetics of the individual stages is elucidated by real-time investigations. As a consequence, the complete synthesis could be optimized and can now be realized in a single day instead of three without any loss in the resulting sample quality.

## 1. Introduction

Rapid developments in the field of nanotechnology have caused a rising interest in plasmonic nanoparticles and, hence, in their synthesis. Thanks to the increasingly numerous advances in optics and photonics, the optical properties of such nanoparticles (NPs) are more and more in the focus of research [1,2]. A notable peculiarity of plasmonic NPs is the phenomenon of the localized surface plasmon resonance (LSPR) occurring at the nanoscale. When excited by incident light, conduction electrons start to oscillate collectively. At the resonant wavelength, such collective oscillations give rise to a characteristic absorption band. The position of the resonance in the UV–VIS spectrum is highly affected by the nanoparticles’ material, size, shape, and chemical environment. These parameters can be tailored according to the given application. For instance, for a broad range of applications, anistropically shaped NPs with sharp edges and well-defined vertices are best suited [3,4,5]. Such NPs are preferable for plasmonic sensor technology due to their higher sensitivity [6,7,8], which is defined as the ratio of observed wavelength shift per refractive index unit. Superstructures [9] or self-assembled layers [10] prepared from gold nanotriangles are utilized in surface-enhanced Raman scattering (SERS) measurements. For quantifying the tip sharpness of such structures, small-angle X-ray analysis can be implemented [11].

The synthesis of plasmonic nanoparticles is often realized by a bottom-up chemical reduction process and results in colloids. Even the simple synthesis of spherical nanoparticles involves numerous parameters, and the reaction kinetics are not thoroughly studied yet [12]. Concerning the synthesis pathway for shape-anisotropic nanoparticles, recent studies have revealed more complex mechanisms with symmetry-breaking events, caused by surfactants and silver or halogenide ions [13,14,15,16,17,18]. Nevertheless, the synthesis of shape-anisotropic nanoparticles is still hampered by a lack of reproducibility.

The strategies to synthesize gold nanoparticles (AuNPs) can be generalized in two categories. Seedless synthesis (one-pot procedure) is faster and less labor consuming [19,20,21,22,23]. However, the seed-mediated approach demonstrates a higher accuracy in tailoring the NPs size due to separated nucleation and growth processes [11,24,25,26]. The major drawback of this method is the instability of the seeds, which is usually compensated by using fast-addition techniques [25,27,28,29]. When reproducing such complex syntheses, one has to keep in mind that parameters like speed and sequence of precursor addition significantly influence the kinetics and, thereby, the shape yield and size distribution of NPs [30]. Recently, Szustakiewicz et al. reported a method involving stable intermediate seeds, in order to avoid the above-mentioned fast-addition step [31]. Furthermore, the authors described the use of a purification procedure based on depletion-forced aggregation, which, in turn, provides a higher shape yield [25,32,33,34]. Nevertheless, the applicability of the method is hindered by an extensive time consumption, as it requires up to 3 days. In the present paper, this synthesis was investigated using time-resolved UV–VIS spectroscopy. This method is commonly used to investigate the kinetics of gold nanoparticle synthesis [35,36,37]. Online monitoring of the optical properties of the reaction mixtures allowed identifying potential for streamlining the protocols without sacrificing yield or reproducibility. These studies resulted in developing a significantly shortened procedure.

## 2. Materials and Methods

### 2.1. Chemicals and Materials

All utilized chemicals were obtained commercially and used without further purification. Tetrachloroauric(III) acid trihydrate (HAuCl_4_ ≥ 99.5%) and L(+)-ascorbic acid (AA, ≥99%) were purchased from Carl Roth GmbH & Co KG (Karlsruhe, Germany). Hexadecyltrimethylammonium chloride (CTAC, >99%) implemented in synthesis procedure was obtained from Molekula Group GmbH (Munich, Germany). Hexadecyltrimethylammonium chloride (CTAC, 25 wt% in water) implemented in purification procedure, sodium borohydride (NaBH_4_, 99.99%), and sodium idode (NaI, ≥99.5%) were purchased from Sigma-Aldrich (Darmstadt, Germany). The solutions were prepared using Millipore water. Prior to use, all glassware and magnetic stirrers were washed with aqua regia (caution: aqua regia is highly toxic and corrosive) and rinsed thoroughly with Millipore water.

### 2.2. Synthesis and Purification of Gold Nanotriangles

Gold nanotriangles (NTs) of various sizes were synthesized following the seed-mediated procedure described by Szustakiewicz et al. [31]. Firstly, initial seeds were obtained by stirring 25 μL of HAuCl_4_ 50 mM solution and 4.7 mL of a CTAC 100 mM solution for 2–3 min and then, under vigorous stirring, by injecting into the reaction mixture 300 μL of a freshly prepared 10 mM NaBH_4_ solution. The mixture had a pale brown color and was left under moderate stirring for 2 h. For the synthesis of intermediate seeds, 16.6 μL of 50 mM HAuCl_4_ and 20 μL of 100 mM AA solutions were mixed with 10 mL of a 25 mM CTAC solution, resulting in a colorless mixture. Then, 100 μL of the as-synthesized initial seed colloid was injected into the reaction mixture under vigorous stirring, kept stirred for 3 more seconds, and was put aside undisturbed for 24 h (or for 40 min in the time-optimized procedure). Within a few seconds, the suspension developed a pink color.

For the final product synthesis, 104 μL of 100 mM AA and 40 μL of 10 mM NaI solutions were mixed with 10.2 mL of 50 mM CTAC. Then, 40 μL of intermediate seed solution (the volume was varied depending on the required size of NTs) was injected rapidly, and the reaction mixture was stirred for 45 s. Further, a pre-mixture (130 μL of 50 mM HAuCl_4_, 40 μL of 10 mM NaI, and 390 μL of deionized water, mixed by hand shaking) was added under vigorous stirring to the reaction vial. The mixture was stirred for a few more seconds and put aside undisturbed for 12 h (or for 2 h in the time-optimized procedure). The suspension color was evolving gradually from red to purple and eventually turned blue within the first minutes. The purification procedure is based on depletion-induced aggregation, which grants the selective precipitation of NTs. The amounts of concentrated CTAC solution added to the crude mixtures depend on the resulting NTs size and, thus, on the volume of intermediate seeds implemented in the growth step. After reaching the desired CTAC concentration (Table S1), the mixture was transferred into a centrifuge tube and left undisturbed for 24 h (or 8 h in the time-optimized procedure). During this time, a small precipitate was formed at the bottom and the supernatant color changed from bluish to pink, showing that mainly isotropic NPs remained in suspension. The supernatant was discarded and the precipitate re-dispersed in water to obtain the purified gold nanotriangle sample.

### 2.3. Characterization Techniques

The colloids of gold nanoparticles were characterized by ultraviolet–visible (UV–VIS) spectroscopy utilizing Thermo Fisher NanoDrop OneC (Waltham, MA, USA) and JASCO V-670 UV–VIS-NIR (Easton, PA, USA) spectrophotometers. In order to measure time-resolved spectra, a single aliquot of a just prepared reaction mixture was instantly transferred to the cuvette and kept under agitation with a magnetic stirrer during the acquisition. The spectra of seed formation were recorded with a NanoDrop spectrometer. A JASCO spectrometer equipped with a cuvette holder allowing for temperature control and stirring (ETC-505S) was used to measure intermediate seed and nanotriangle reaction mixtures.

Scanning electron microscopy (SEM) images were taken using a JEOL FE-SEM JSM-7900F (Akishima, Japan). The probes were prepared following the modified literature technique [38]. An amount of 1.8 mL of the AuNT suspension containing ca. 5 × 10^−7^ mol of Au^0^ (0.278 mM; A_400_ = 0.667) was centrifuged and re-dispersed in 0.6 mL of 1 mM CTAC. To ensure the required surfactant concentration for optimal imaging, the NPs were centrifuged and re-dispersed in 1 mL of 0.1 mM CTAC (resulting in 0.5 mM Au^0^). Finally, the samples were centrifuged and re-dispersed in 0.12 mL of 0.1 mM CTAC, and 2.5 μL of the colloid was cast on a silicon substrate.

## 3. Results

**Scheme 1:**
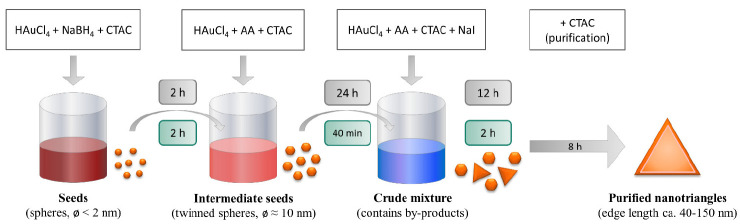
General overview of the synthetic procedure. The time intervals proposed by Szustakiewicz et al. [31] are highlighted in gray, the intervals of the proposed time-optimized procedure are highlighted in green.

The synthesis of gold nanotriangles is represented in Scheme 1. To study the evolution of AuNPs during the synthesis, UV–VIS spectra at the stages of seed formation, intermediate seed, and nanotriangle growth were obtained by online monitoring. A time-optimized procedure with shortened intervals was proposed based on the analysis of each step. Moreover, the applicability of intermediate seeds within one week of storage is shown in Supplementary Materials (Figure S1).

### 3.1. Kinetic Studies

#### 3.1.1. Seed Formation

Upon mixing the aqueous solution of HAuCl_4_ with CTAC, the color turns to a brighter yellow, indicating the formation of the complex [39]:

CTA^+^ + AuCl_4_^−^ → CTA—AuCl_4_ (1)

Further on, the Au^3+^ in complex is reduced to Au^0^ by adding NaBH_4_:

8CTA—AuCl_4(aq)_ + 3NaBH_4__(aq)_ + 6H_2_O_(l)_ → 8Au_(s)_ + 3NaBO_2(aq)_ + 8CTA—Cl_(aq)_ + 24HCl_(aq)_ (2)

Simultaneously, the hydrolysis of excessive NaBH_4_ takes place [40,41]:

NaBH_4(aq)_ + 2H_2_O_(l)_ → NaBO_2(aq)_ + 4H_2(g)_ (3)

The generation of H_2_ induces the formation of bubbles, resulting in the inhomogeneity of the reaction mixture. Hence, incubation of 2 h is usually required for the complete reaction of the remaining NaBH_4_.

**Figure 1:**
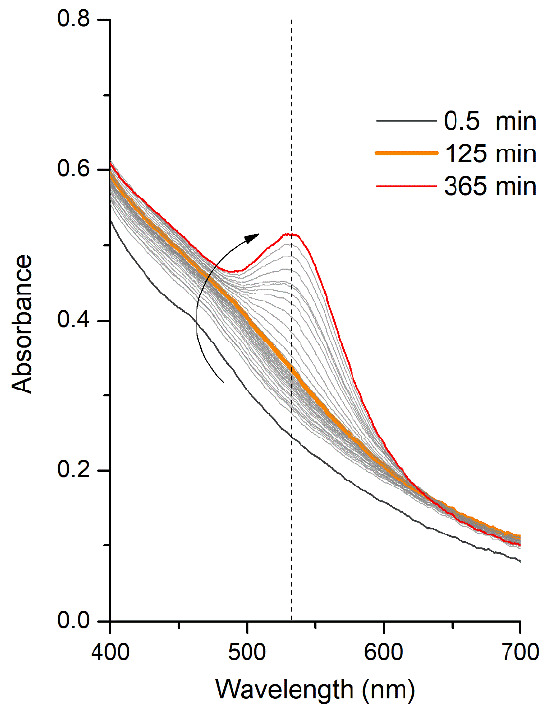
UV–VIS spectra showing the process of seed formation. The orange bold line indicates the beginning of seed overgrowth at 125 min. The selected wavelength position at 532.5 nm is marked by a dotted line. The arrow indicates the time evolution.

At first, seeds were synthesized, and the obtained time-resolved UV–VIS spectra investigated, for assessing within which period they are applicable for the next stages. The color of the reaction mixture changed from pale yellow to brown instantly after the injection of NaBH_4_ due to its high reactivity. Further changes in optical properties could only be resolved with UV–VIS spectrometry, the data of which are shown in Figure 1; a 3D visualization can be found in Figure S2. The last spectrum was measured at 365 min after the reaction start. In this curve, the LSPR peak can be found at 532.5 nm, demonstrating the formation of spherical particles with a diameter of more than 2 nm [42,43]. Seeds smaller than 2 nm in diameter do not show the LSPR band in the UV–VIS spectrum and are preferable for the synthesis, while the use of larger seeds may affect the synthesis efficiency [44,45,46,47,48,49]. Therefore, the pronounced LSPR peak represents an indicator of an undesired seed overgrowth, and the next stage of the synthesis should be carried out before it occurs. The values of A_400_ correlate with Au^0^ concentration [25,50] and, therefore, serve as a tool for tracing the NP formation process from Au^3+^ precursor.

**Figure 2:**
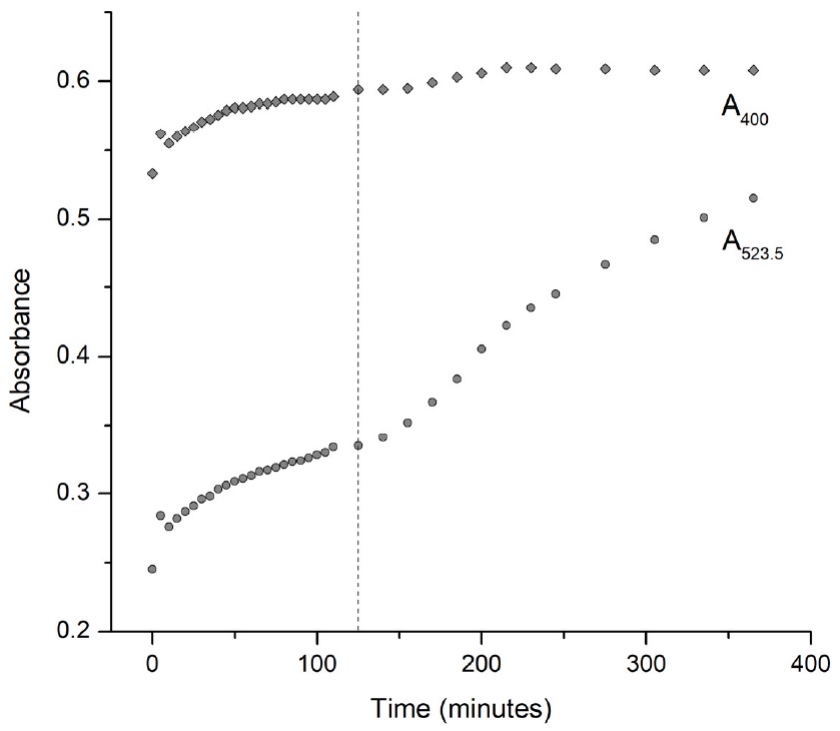
The kinetic curves plotted for two positions in the UV–VIS spectrum: absorbance at 400 nm (Au^0^) and at 532.5 nm (spheres >2 nm in diameter). The dashed line refers to the seed overgrowth at 125 min after the reaction start.

Taking into the account the above-mentioned statements, the kinetic curves presented in Figure 2 were plotted using two spectral positions: absorbance at 400 nm and at 532.5 nm. Within 2 h, the intensity at these wavelengths increased from 0.533 to 0.594 and from 0.245 to 0.335, respectively, pointing to the formation of seeds. After 125 min, the A_400_ values did not change significantly, confirming that the HAuCl_4_ in the reaction mixture had been completely reduced to form the nanoparticles. On the other hand, an increase of A_532.5_ values up to 0.515 started to occur at the same time, indicating an overgrowth of seeds caused by their coalescence, which should be minimized for optimal synthesis results.

Sodium borohydride is a highly reactive reducing agent implemented for instant Au^3+^ → Au^0^ transformation during the seed formation. Once it has abreacted, the synthesis should only be continued with a milder reducing agent, such as ascorbic acid, in order to avoid the risk of coalescence of NPs during the growth. Considering the discussion of the kinetic curves (and the behavior following the 125 min time point), the optimal point to proceed with the next stage is after 2 h of reaction time. The seeds should be used immediately for growing intermediate seeds in order to avoid the overgrowth processes, which might undesirably broaden the size distribution of the resulting samples.

#### 3.1.2. Intermediate Seed Growth

Firstly, the pale-yellow solution of HAuCl_4_ is mixed with AA, resulting in a colorless solution due to the Au^3+^ → Au^+^ transformation. During this process, ascorbic acid is oxidized to dehydroascorbic acid:

CTA—AuCl_4(aq)_ + C_6_H_8_O_6(aq)_ → CTA—AuCl_2(aq)_ + C_6_H_6_O_6(aq)_ + 2HCl_(aq)_ (4)

Once the seed suspension is injected into the reaction mixture, the color turns pink due to the NPs growth:

2CTA—AuCl_2(aq)_ + C_6_H_8_O_6(aq)_ → 2Au_(s)_ + C_6_H_6_O_6(aq)_ + 2CTA—Cl_(aq)_ + 2HCl_(aq)_ (5)

The reduction of Au^+^ onto the growing nanoparticle occurs faster than new nucleation events, ensuring a narrow size distribution and high shape yield. This seeding process takes place due to the surface of gold nanoparticles (initial seeds) serving as a catalyst and, thus, lowering the reduction potential of gold [46].

**Figure 3:**
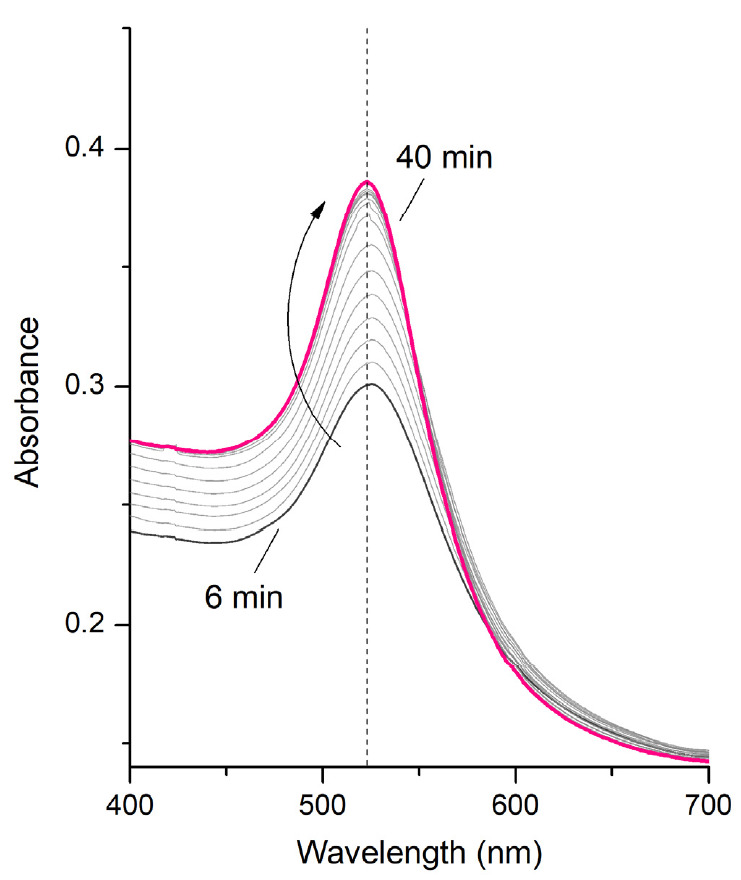
UV–VIS spectra showing the process of intermediate seed growth. The selected wavelength position at 523 nm is marked by a dotted line. The arrow indicates the time evolution.

The changes in optical properties during the process were recorded by means of UV–VIS spectroscopy: a 3D visualization of the observed behavior can be found in Figure S3a. The spectra obtained by online monitoring are shown in Figure 3. According to the proposed seed-mediated growth mechanism, the LSPR peak should demonstrate the progressive red-shift due to the increase of NPs size. In fact, the slight blue-shift is observed from 525 nm (at 6 min) to 523 nm (at 40 min). This can be explained by the interaction of gold precursor species with the surface of nanoparticle, which causes the shift of the band position to higher wavelengths [50]. Upon the reduction process, the amount of HAuCl_4_ decreases, lowering the effect of the above-mentioned interaction and shifting the LSPR peak “back” to the lower wavelengths [36]. The last spectrum was measured at 105 min after the reaction started (Figure S3b). It has the same maximum position at 523 nm, which relates to the spherical particles with a diameter of ca. 10 nm [31]. It is assumed that intermediate seeds can be used as soon as their growth has finished. Therefore, steady intensity values of the LSPR peak can be an indicator of the completed growth, so that the next stage of the synthesis could be carried out. The values of A_400_ were used to trace the reaction process of forming the NPs from Au^3+^ precursor.

**Figure 4:**
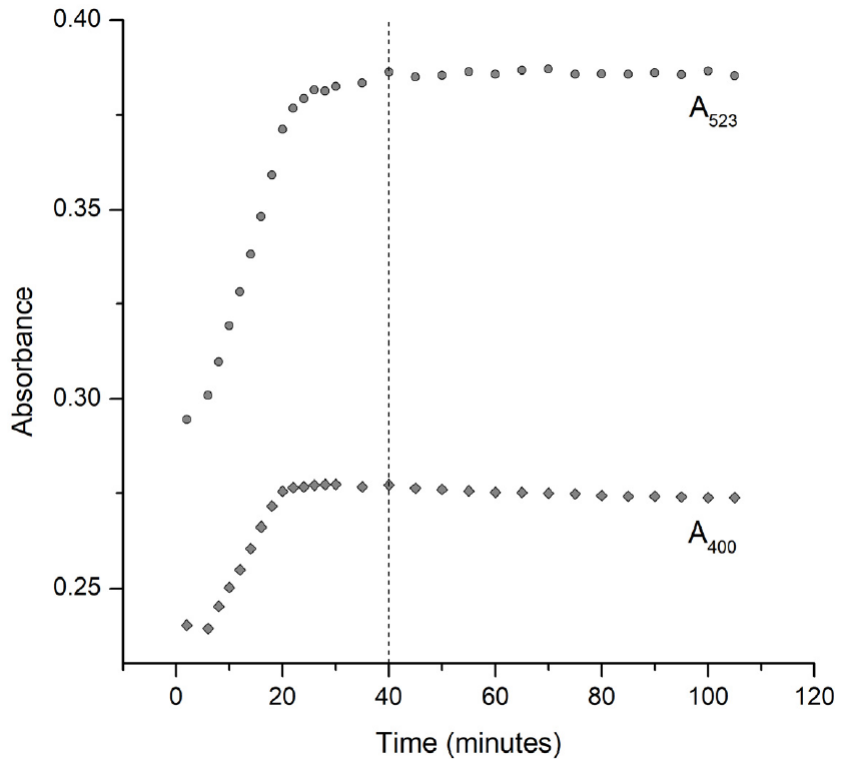
The kinetic curves for two positions in UV–VIS spectra: absorbance at 400 nm (Au^0^) and at 523 nm (spheres ca. 10 nm in diameter). The dashed line indicates the growth process completion 40 min after the reaction start.

Taking into account the statements above, the kinetic curves presented in Figure 4 were plotted using absorbance measurements at two positions in the spectra: at 400 nm and at 523 nm. Within 40 min after the beginning of the reaction, the intensity at these wavelengths increased from 0.240 to 0.277 and from 0.295 to 0.386, respectively, showing the growth of seeds into larger spherical gold particles. Subsequently, the A_400_ and A_523_ values did not change significantly, proving that the entire HAuCl_4_ in the reaction mixture had already been reduced to grow intermediate seeds.

UV–VIS spectra of intermediate seeds measured 3 days after the reaction start show that there are no significant changes in the optical properties within this period (Figure S3b). Presumably, an incubation time of 40 min is sufficient for complete HAuCl_4_ reduction with AA, so to continue with the next stage of the synthesis.

#### 3.1.3. Nanotriangle Growth

The chemical reaction for the formation of nanotriangles is identical to the intermediate seed growth process and is already described by the Equations (4) and (5). The main difference is the addition of iodide, which is known to be an essential shape-directing reagent. The most widespread interpretation of its influence was given by Mirkin and co-workers: they proposed that iodide ions can strongly and selectively bind to the Au (111) facet, favoring the formation of nanotriangles [18].

**Figure 5:**
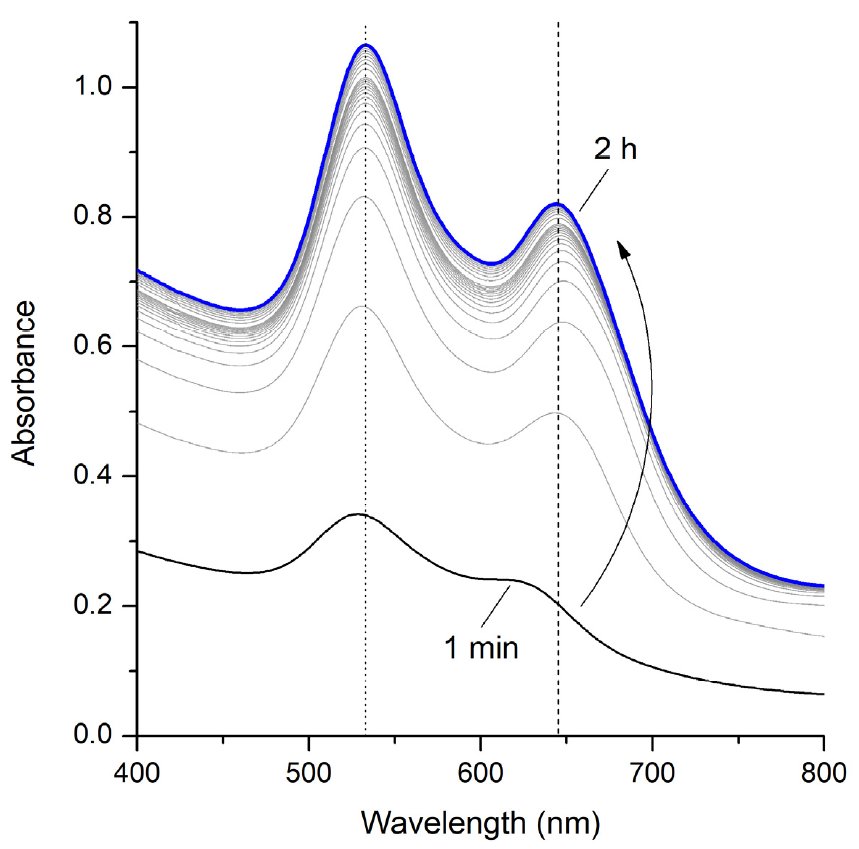
UV–VIS spectra showing the process of nanotriangle growth, selected wavelength positions at 533 and 645 nm are marked by dotted lines. The arrow indicates the time evolution.

Once all the chemicals were mixed, the color of the colloid changed gradually from red to purple within the first minutes and eventually turned blue. This color evolution was studied by online UV–VIS monitoring: the obtained spectra are shown in Figure 5, while the 3D visualization of the process can be found in Figure S4a. At first, the formation of isotropic products with LSPR peak at ca. 530 nm took place. Subsequently the fraction of grown nanotriangles also contributed to the resulting spectrum with a LSPR peak at 645 nm. These peaks can be clearly identified in the last spectrum, measured 300 min after the reaction start (Figure S4b). Supposedly, the reaction mixture can be purified as soon as the NPs’ growth has finished. Therefore, steady intensity values of the LSPR peaks would be an indicator for completed growth, so that the purification procedure can be carried out. The values at 400 nm were used to trace the reaction process of forming the NPs from Au^3+^ precursor.

**Figure 6:**
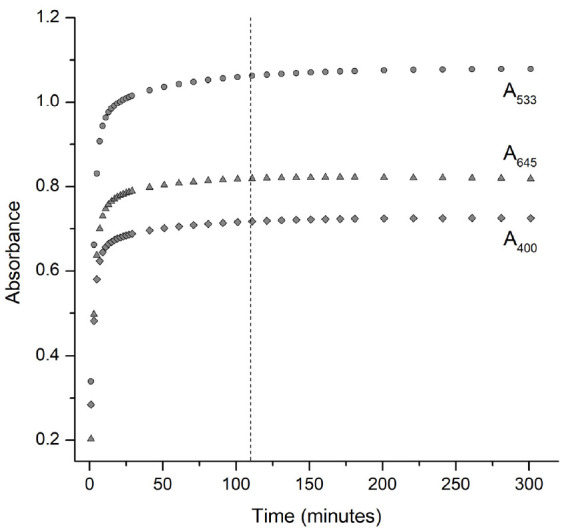
Kinetic curves representing the absorbance values at 400 nm (Au^0^), at 533 nm (by-products), and at 645 nm (nanotriangles). The dashed line is indicating the growth process completion after 2 h.

Based on the above-mentioned remarks, the kinetic curves presented in Figure 6 were plotted using three wavelength positions: absorbance at 400, 533, and 645 nm. Within 2 h after the reaction start, the intensity at these wavelengths increased from 0.285 to 0.717, from 0.338 to 1.063, and from 0.203 to 0.818, respectively, showing the growth of intermediate seeds into two fractions of particles: isotropic byproducts (as evidenced by the peak at 533 nm) and nanotriangles (peak at 645 nm). Subsequently, these values did not change significantly, indicating that the entire HAuCl_4_ in the reaction mixture had already been reduced.

UV–VIS spectra of the crude mixture measured 1 day after the reaction start show that there are no significant changes in the optical properties within this period (Figure S4b). Presumably, an incubation time of 2 h for completing the HAuCl_4_ reduction is sufficient to continue with the purification step.

### 3.2. Original and Time-Optimized Synthesis Procedures

Kinetic studies allowed for determining reasonable time intervals required for completing each step of the synthesis. The summary is shown in Table 1 in comparison to the procedure originally described by Szustakiewicz et al. [31]. Following the optimized protocol, the entire procedure could be significantly shortened from three days to one.

To confirm that the proposed optimization does not lead to a quality loss of the resulting samples, two parallel experiments were carried out following each of the mentioned procedures. From a single seed batch, two intermediate seed samples were produced. The UV–VIS spectrum of seeds was typical for gold nanoparticles with diameters of less than 2 nm (Figure S5a). The spectra of intermediate seeds demonstrated no significant differences between each other (Figure S5b). This proves that the synthesis is fairly reproducible, and any differences in resulting sample properties would be caused only by differences in time intervals of each procedure.

**Figure 7:**
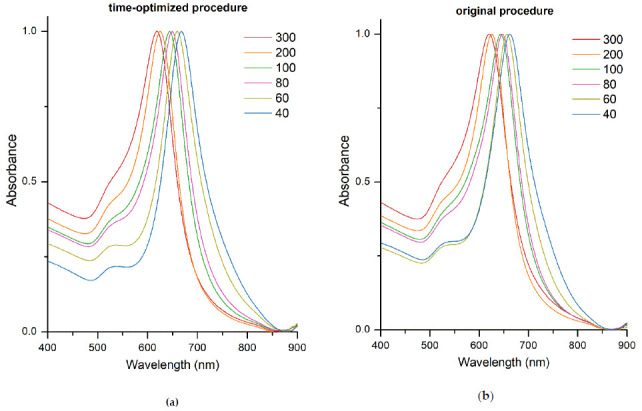
UV–VIS spectra of purified gold nanotriangles synthesized after time-optimized (**a**) and after original procedure (**b**). The legends indicate the volume (μL) of intermediate seeds used for each sample.

The purified samples of nanotriangles were examined with UV–VIS spectroscopy and SEM imaging. The obtained UV–VIS spectra exhibit no significant differences in curve lineshape, although a shift of the NTs band can be observed from 620 nm to ca. 670 nm depending on the volume of intermediate seeds utilized and, thus, on the size of the resulting triangles. For a more detailed analysis, the LSPR peaks were determined (Table 2) and the data normalized (Figure 7).

The sizes of nanotriangles were estimated using the LSPR peak positions (Table S2). The calculated values are quite similar for samples grown from the same amount of intermediate seeds, independently from the followed reaction time intervals. The lowest differences, within 1 nm, are determined for the smallest particles (300, 200, and 100 μL of intermediate seeds). For the larger nanotriangles (80, 60, and 40 μL of intermediate seeds), the distinction in size values is higher, but does not exceed 4 nm.

Another considerable characteristic that can be derived from the UV–VIS data is the indirect information about the shape yield: it is known that the band at ca. 530 nm corresponds to the isotropic by-products formed during the synthesis [25,44]. The differences of the intensities at 530 nm for triangles of the same size do not exceed 0.1 a.u., proving that the shape yield does not depend on the procedure followed (Table S3). A similar conclusion can be made about the size distribution of the NTs indirectly characterized by the LSPR peak width at ca. 650 nm (Table S4).

**Figure 8:**
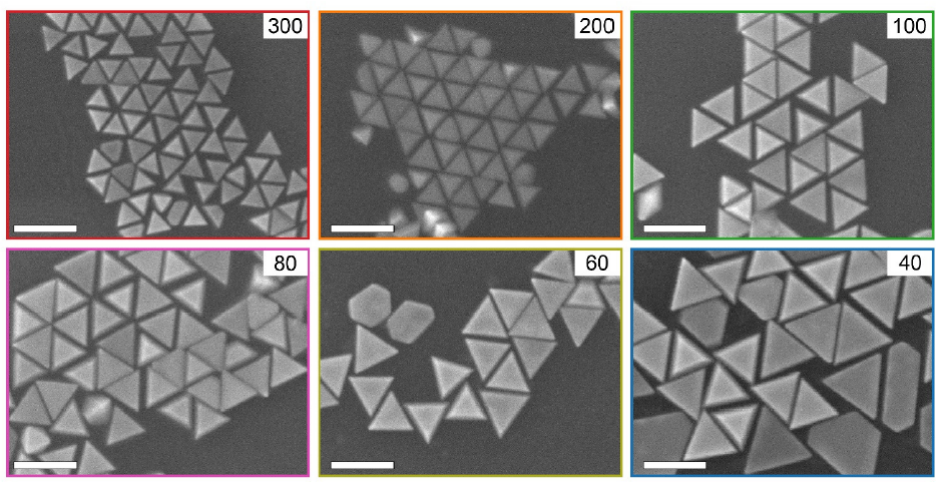
SEM images of purified gold nanotriangles synthesized after the time-optimized procedure. Labels indicate the volume (μL) of intermediate seeds used for each sample. Scale bars are 100 nm.

The SEM images of purified samples reveal the successful formation of particles with triangular shape (Figure 8). No significant differences are noted during the comparison with the images of samples obtained after the original procedure (Figure S6).

The average edge lengths (Table 3) were obtained from statistical analysis of SEM data and are in a good agreement with findings obtained from the UV–VIS measurements. The histograms showing the size distribution can be found in Figures S7 and S8.

Overall, the nanotriangle samples demonstrate comparable quality characteristics such as size distribution and shape yield, regardless of the reaction time intervals followed during the synthetic procedure.

## 4. Conclusions

Kinetic studies of the gold nanotriangles seed-mediated synthesis are presented. Time-resolved UV–VIS measurements of the reaction mixture were utilized as a powerful tool for an online monitoring of the synthesis evolution at each step. The obtained data, in combination with the knowledge of the occurring chemical processes, allowed to rationally determine the time intervals required for a successful reproduction of the procedure. This resulted in setting up a modified protocol providing the purified samples one day after the synthesis was started. In comparison, the work published earlier [31], although describing the same synthetic route, comes with a major limitation: the overall synthesis requires three days, so that the final product can be only collected on the fourth day. To demonstrate that the proposed alteration does not affect the quality of the resulting samples, two parallel experiments, according to each protocol, were performed. The UV–VIS spectra and SEM images of nanotriangles revealed major characteristics, such as size distribution and shape yield, to be comparable for both procedures.

The resulting modified protocol represents an example of how spectroscopic approaches can be utilized for determining the critical durations of key synthesis steps, and for exploring the potential of significantly shortening the synthesis time without losing robustness and reproducibility. Additionally, it enables streamlining of the targeted synthesis, and may have a broader applicability in order to improve the synthesis of anisotropic plasmonic nanoparticles for future applications in fields like bioanalytics and catalysis.

## Figures and Tables

**Table 1:** Optimization of time consumption for the nanotriangle synthesis.

Step	Time-Optimized Procedure	Original Procedure
Seeds	2 h	2 h
Intermediate seeds	40 min	24 h
Triangles	2 h	12 h
Purification	8 h	24 h
Total:	1 day	3 days

**Table 2:** Comparison of LSPR peak position (λ_max_, nm) for the samples obtained after the time-optimized and original procedures.

Volume of Intermediate Seeds, μL	Time-Optimized Procedure	Original Procedure
300	619	620
200	625	626
100	644	643
80	649	647
60	660	656
40	668	662

**Table 3:** Average edge lengths (nm ± SD) for the samples obtained after the time-optimized and original procedures.

Volume of Intermediate Seeds, μL	Time-Optimized Procedure	Original Procedure
300	37 ± 3	37 ± 3
200	45 ± 4	44 ± 4
100	58 ± 5	58 ± 5
80	62 ± 7	63 ± 6
60	76 ± 13	70 ± 7
40	82 ± 14	79 ± 8

## Data Availability

Raw data utilized within this article were generated at Leibniz-Institute of Photonic Technology, Jena, Germany. The data presented in this study are available on request from the corresponding author (W.F.).

## Supplementary Materials

The following are available online at https://www.mdpi.com/article/10.3390/nano11041049/s1, Table S1: CTAC concentrations used for purifying the samples with nanotriangles of different sizes; Figure S1: (a) UV–VIS spectra of intermediate seeds within 7 days of storage at RT, (b) UV–VIS spectra of gold nanotriangle grown from 100 µL of aged intermediate seeds; Figure S2: 3D visualization of changes in optical properties during seed formation step; Figure S3: (a) 3D visualization of changes in the optical properties during the intermediate growth step, (b) UV–VIS spectra of intermediate seeds measured at 40 min, 105 min, and 3 days after the reaction start; Figure S4: (a) 3D visualization of changes in the optical properties during the nanotriangle growth step. (b) UV–VIS spectra of crude reaction mixture measured at 2 h, 5 h, and 1 day after the reaction start; Figure S5: UV–VIS spectra of seeds (a) and intermediate seeds (b) used in the synthesis of gold nanotriangles; Table S2: Estimated edge lengths from UV–VIS data for the samples obtained after the time-optimized and original procedures; Table S3: Comparison of LSPR peak intensity at 530 nm (a.u.) for evaluation of by-products content in purified samples; Table S4: Comparison of UV–VIS curves FWHM (nm) for evaluation of size distribution in nanotriangle purified samples; Figure S6: SEM images of purified gold nanotriangles synthesized after the original procedure; Figure S7: Size distribution histograms obtained with statistical analysis of SEM images for samples synthesized after the time-optimized procedure; Figure S8: Size distribution histograms obtained with statistical analysis of SEM images for samples synthesized after the original procedure.
