# Supplementary material for: Time Optimization of Seed-Mediated Gold Nanotriangle Synthesis Based on Kinetic Studies
Source: Nanomaterials (Basel). 2021 Apr 20;11(4):1049. doi: 10.3390/nano11041049 (PMC8073722; doi:10.3390/nano11041049)
Supplement: Supplementary file 1 [file nanomaterials-11-01049-s001.zip › nanomaterials-1145454-supplementary.pdf]

nanomaterials

Supplementary Materials

Time Optimization of Seed-Mediated Gold Nanotriangle Synthesis Based on Kinetic Studies

Ekaterina Podlesnaia, Andrea Csáki and Wolfgang Fritzsche *

Department of Nanobiophotonics, Leibniz Institute of Photonic Technology (IPHT), Albert-Einstein-Straße 9, 07745 Jena, Germany; ekaterina.podlesnaia@leibniz-ipht.de (E.P.); andrea.csaki@leibniz-ipht.de (A.C.)
* Correspondence: wolfgang.fritzsche@leibniz-ipht.de; Tel.: +49-(0)3641-206-304

Table S1. CTAC concentrations used for purifying the samples with nanotriangles of different sizes.

Volume of Intermediate Seeds, µL	CTAC Concentration, mM
300	450
200	300
100	200
80	175
60	125
40	100

The applicability of intermediate seeds within one week of aging was studied. Szustakiewicz et al. have demonstrated the stability of intermediate seeds within 7 days using UV–Vis spectroscopy [1]. Herein we report an extension to these studies with resulting nanotriangle evaluation. Each sample was grown from 100 µL of intermediate seeds and stored for 1, 2, 3, 4 and 7 days at room temperature protected from light. UV–Vis spectra revealed now significant differences in optical properties of intermediate seeds during storage (Figure S1a). In spectra of nanotriangles the LSPR peak position varies within 15 nm (Figure S1b). Nevertheless, the size distribution (width of the curves) and shape yield (intensity of by-products' peak at ca. 530 nm) are comparable for every sample. This data demonstrates that intermediate seeds can be used for the nanotriangle growth within at least one week without any quality loss.

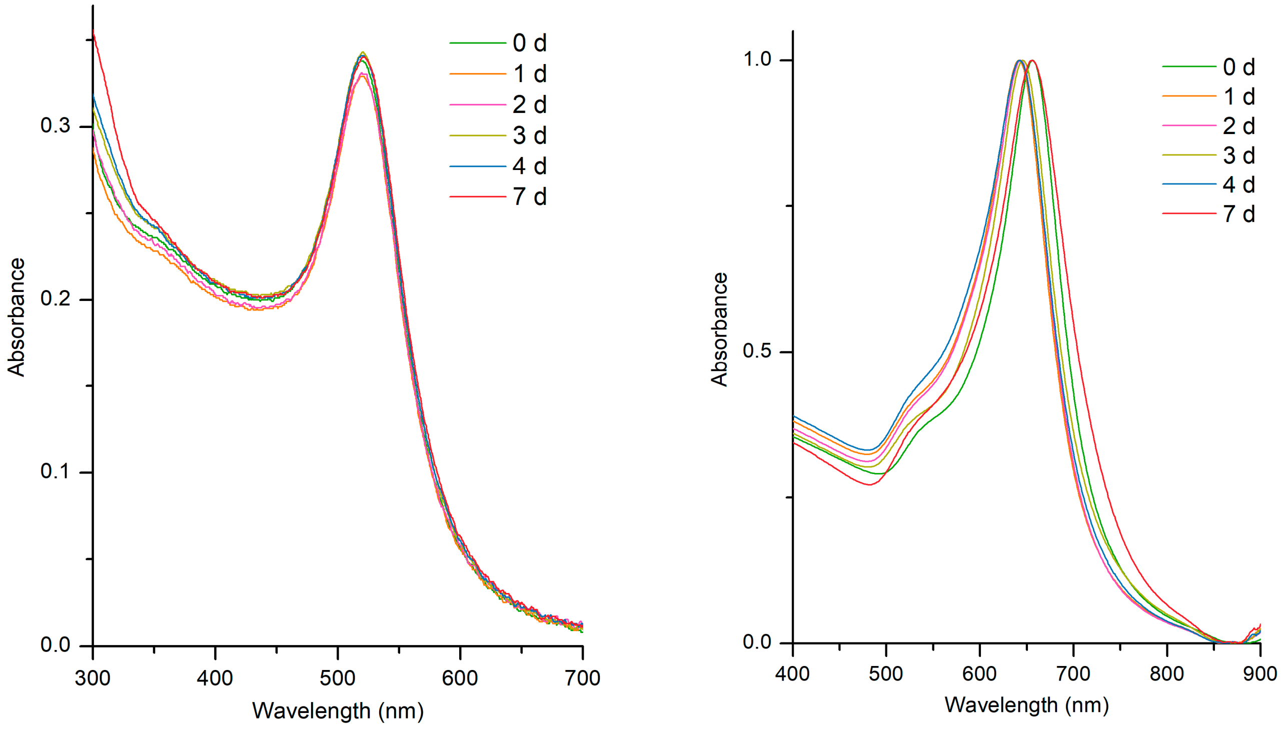

(a) **(b)**

Figure S1. UV–Vis spectra of intermediate seeds within 7 days of storage at RT (**a**) and gold nanotriangle grown from 100 μL of aged intermediate seeds (**b**). The legends indicate the aging time (days) of intermediate seeds used for each measurement / sample. “0 d” corresponds to the sample obtained with using fresh intermediate seeds.

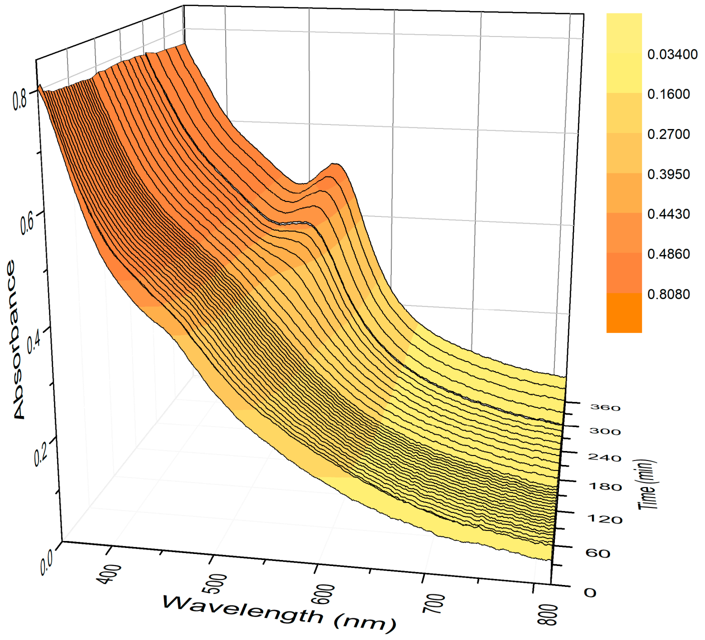

Figure S2. 3D visualization of changes in optical properties during seed formation step.

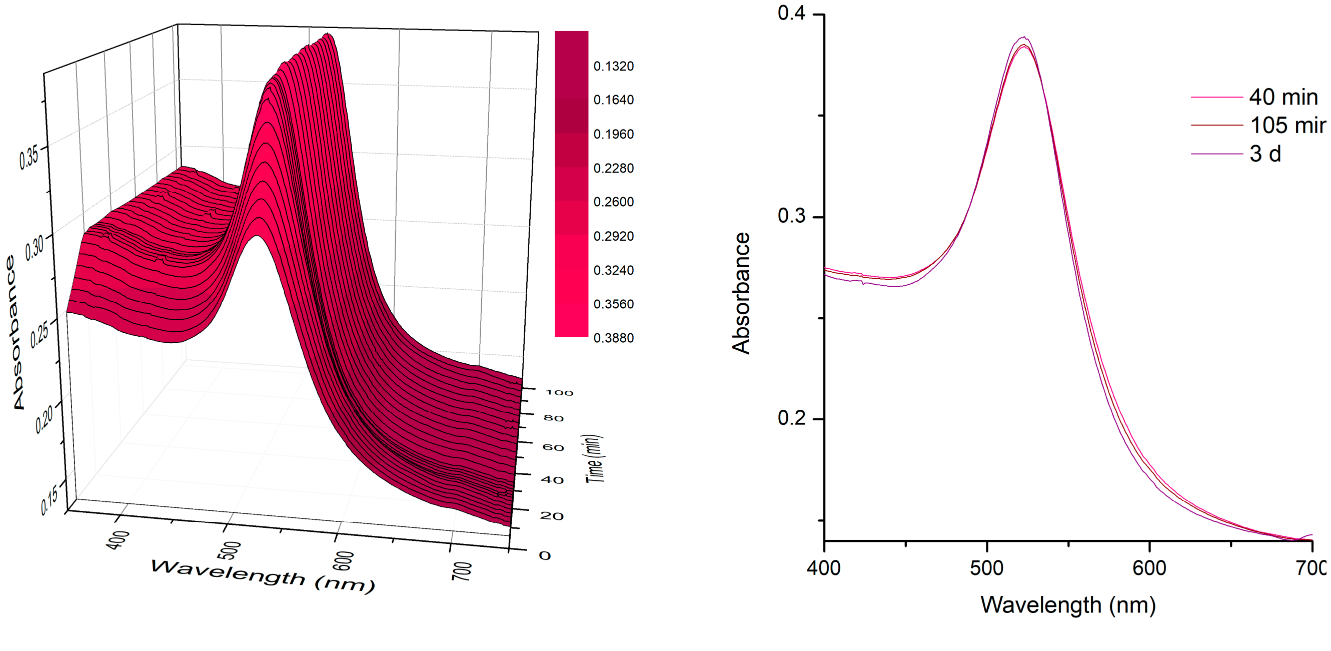

(**a**) (**b**)

Figure S3. (**a**) 3D visualization of changes in the optical properties during the intermediate growth step. (**b**) UV–Vis spectra of intermediate seeds measured at 40 min, 105 min, and 3 days after the reaction start proving no significant changes in optical properties within this period.

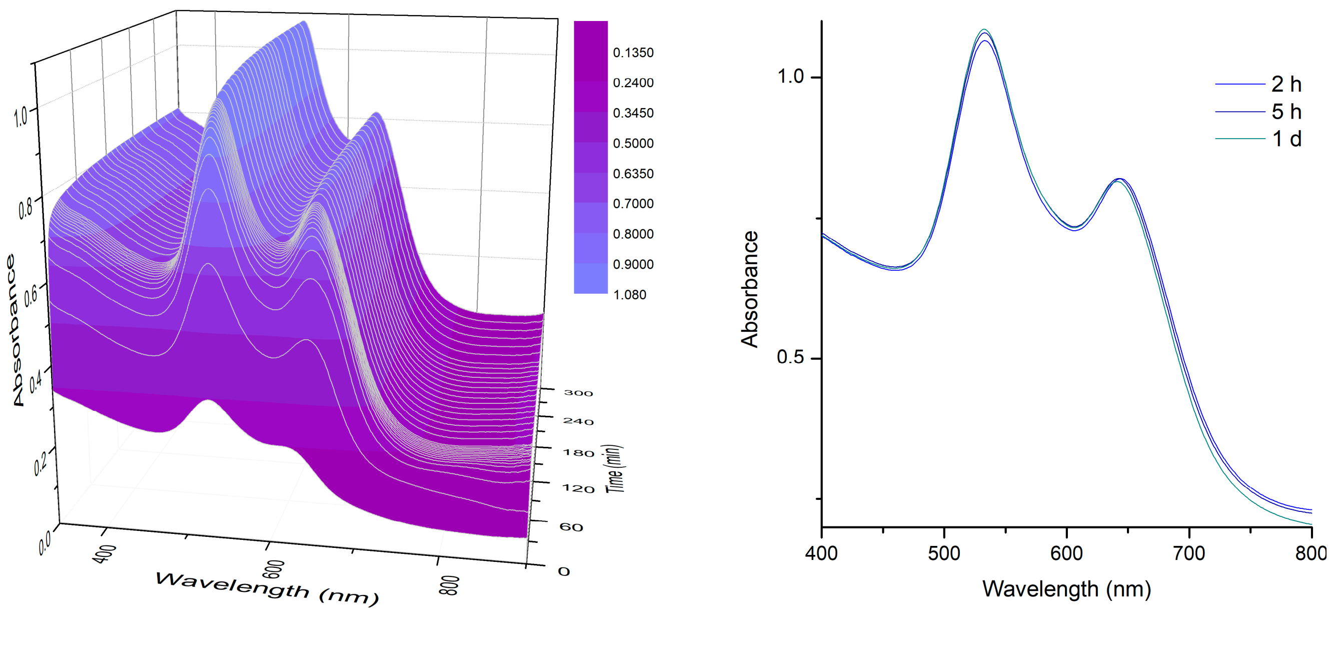

(a) **(b)**

Figure S4. (**a**) 3D visualization of changes in the optical properties during the nanotriangle growth step. (**b**) UV–Vis spectra of crude reaction mixture measured at 2 h, 5 h, and 1 day after the reaction start proving no significant changes in optical properties within this period.

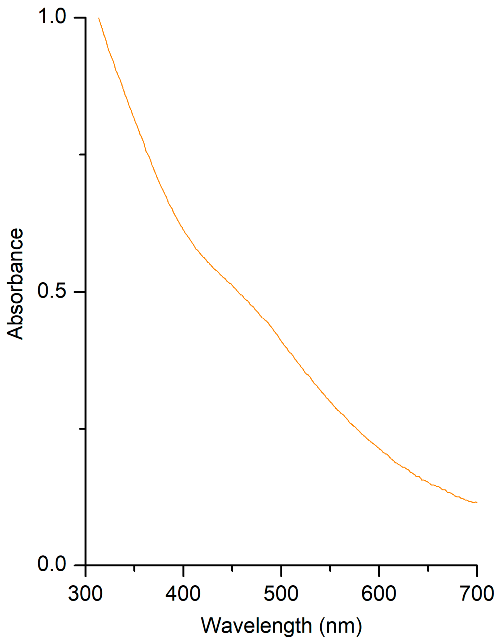

(**a**)

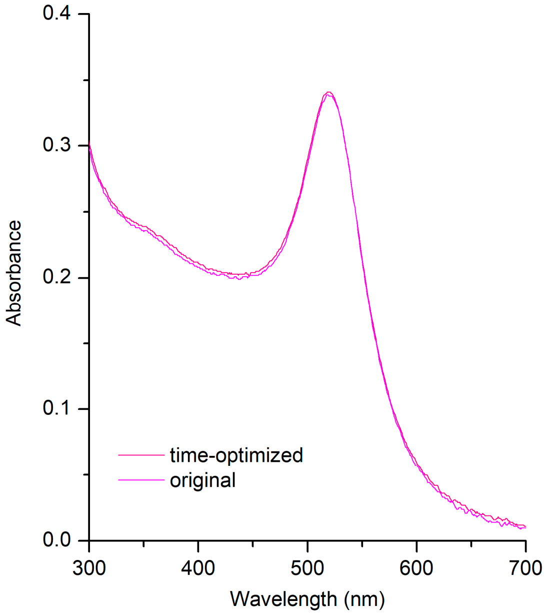

(**b**)

Figure S5. UV–Vis spectra of seeds (**a**) and intermediate seeds (**b**) used in the synthesis of gold nanotriangles. The legend describes in which procedure each of the intermediate seed sample was utilized.

The linear function was extracted from the data published by Szustakiewicz et al. [1] for estimating an average edge length of triangles (L, nm) from the LSPR peak position (λ_{max}, nm): $L = 0.6361 \times \lambda_{max} - 348.76$; the calculated values are presented in Table S2.

Table S2. Estimated edge lengths (L, nm) from UV–Vis datafor the samples obtained after the time-optimized and original procedures.

Volume of Intermediate Seeds, µL	Time-Optimized Procedure	Original Procedure
300	45.0	45.6
200	48.8	49.5
100	60.9	60.3
80	64.1	62.8
60	71.1	68.5
40	76.2	72.4

Table S3. Comparison of LSPR peak intensity at 530 nm (a.u.) for evaluation of by-products content in purified samples.

Volume of Intermediate Seeds, µL	Time-Optimized Procedure	Original Procedure
300	0.51	0.52
200	0.44	0.45
100	0.38	0.41
80	0.36	0.38
60	0.29	0.28
40	0.22	0.29

Table S4. Comparison of UV–Vis curves FWHM (nm) for evaluation of size distribution in nanotriangle purified samples.

Volume of Intermediate Seeds, μL	Time-Optimized Procedure	Original Procedure
300	134	140
200	102	105
100	98	112
80	97	105
60	91	88
40	92	100

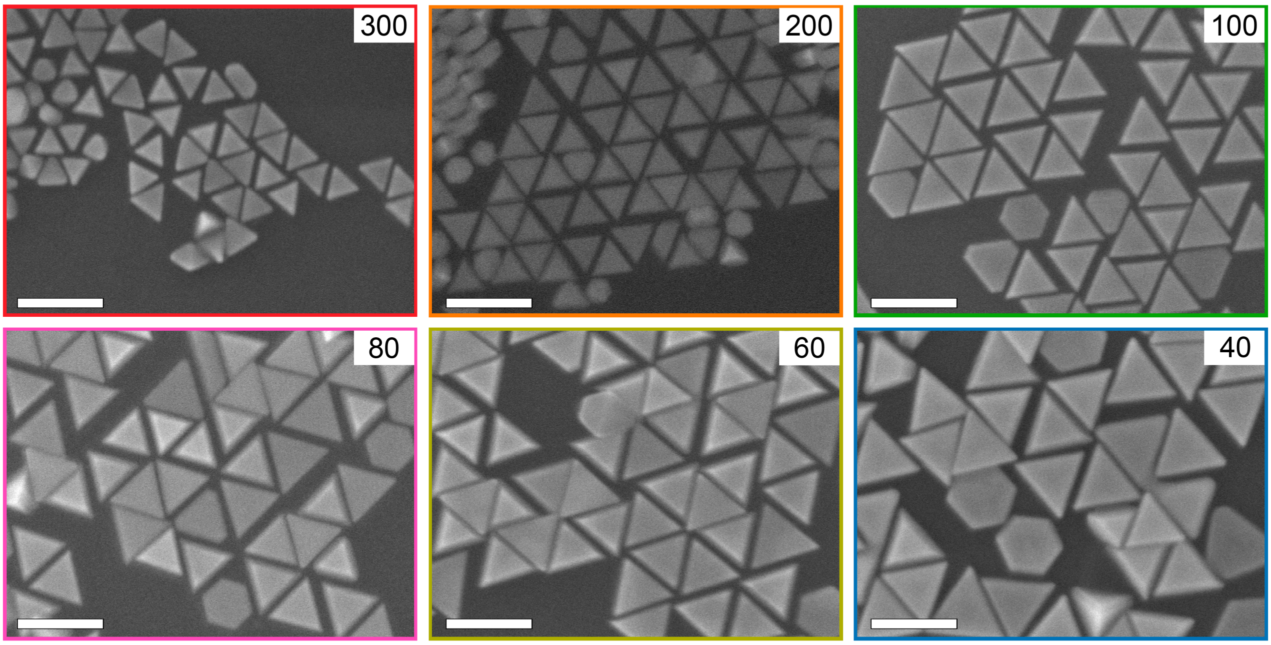

Figure S6. SEM images of purified gold nanotriangles synthesized after the original procedure. Labels indicate the volume (μL) of intermediate seeds used for each sample. Scale bars are 100 nm.

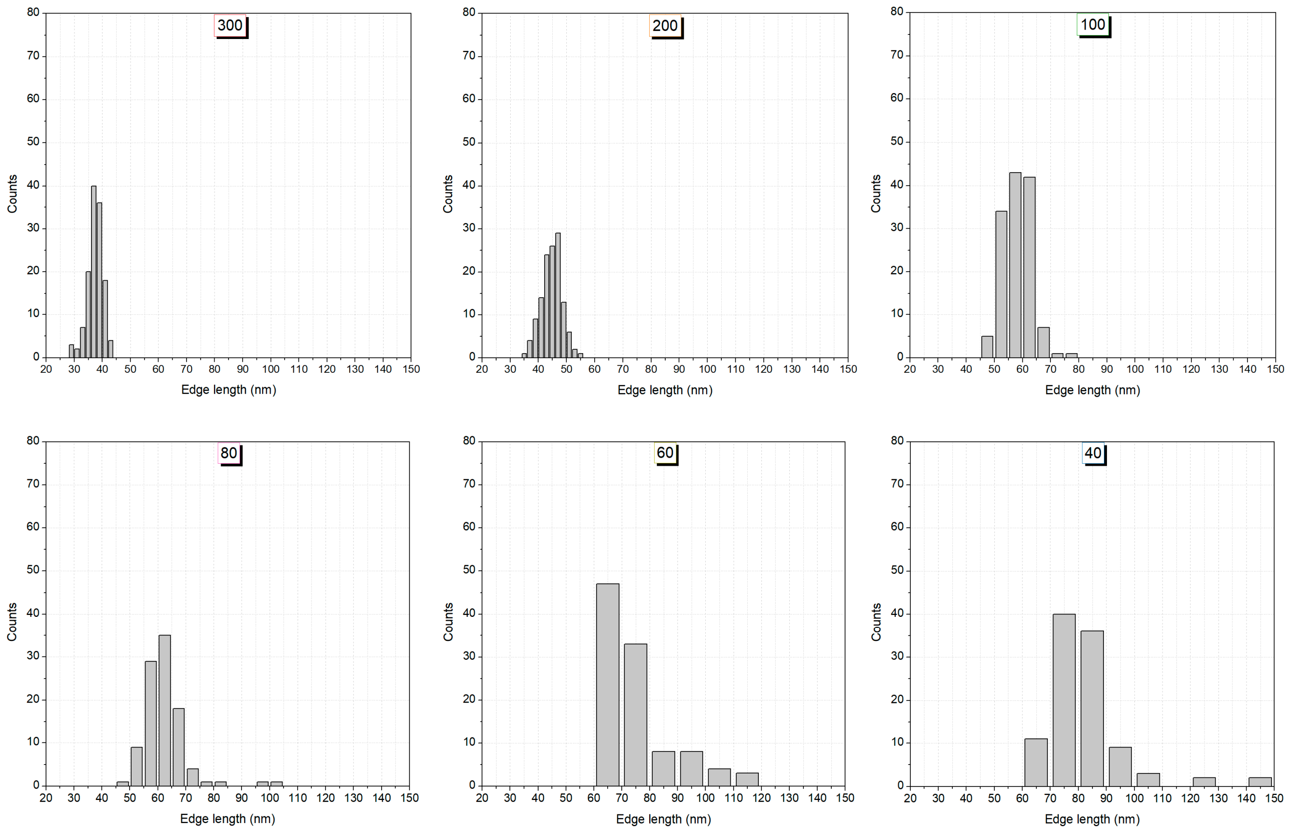

Figure S7. Size distribution histograms obtained with statistical analysis of SEM images for samples synthesized after the time-optimized procedure. At least 100 (and up to 130) NPs were counted from 1–2 images. Labels indicate the volume (μL) of intermediate seeds used for each sample.

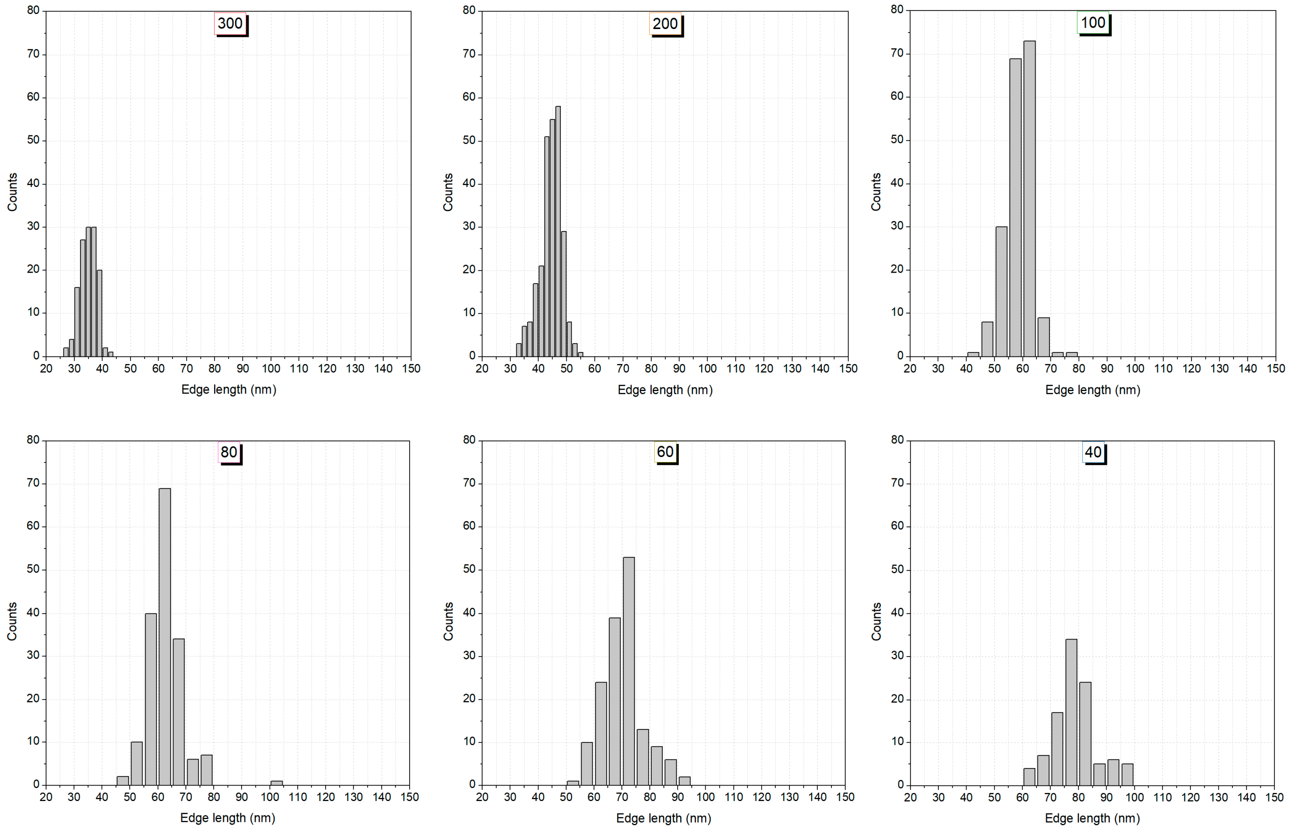

Figure S8. Size distribution histograms obtained with statistical analysis of SEM images for samples synthesized after the original procedure. At least 100 (and up to 260) NPs were counted from 1–2 images. Labels indicate the volume (μL) of intermediate seeds used for each sample.

Reference

1. Szustakiewicz, P.; González-Rubio, G.; Scarabelli, L.; Lewandowski, W. Robust Synthesis of Gold Nanotriangles and their Self-Assembly into Vertical Arrays. *Chem. Open* **2019**, *8*, 705–711, doi:10.1002/open.201900082.
